# The *Great Recession* and Fertility in Europe: A Sub-national Analysis

**DOI:** 10.1007/s10680-020-09556-y

**Published:** 2020-04-03

**Authors:** Anna Matysiak, Tomáš Sobotka, Daniele Vignoli

**Affiliations:** 1 Faculty of Economic Sciences, University of Warsaw, ul. Długa 44/50, 00-241 Warsaw, Poland; 2 Wittgenstein Centre for Demography and Global Human Capital (IIASA, VID/ÖAW, WU), Vienna Institute of Demography/Austrian Academy of Sciences, Vordere Zollamtsstrasse 3, 1030 Vienna, Austria; 3 University of Florence, Viale G.B. Morgagni 59, 50134 Florence, Italy

**Keywords:** Fertility, Economic recession, Economic uncertainty, Europe, Regional differences, Unemployment

## Abstract

This study investigates how the changes in labour market conditions and economic growth were associated with fertility before and during the *Great Recession* in Europe in 2002–2014. In contrast to previous studies, which largely concentrated at the country level, we use data for 251 European regions in 28 European Union (EU) member states prior to the withdrawal of the United Kingdom in January 2020. We apply three-level growth-curve model which allows for a great deal of flexibility in modelling temporal change while controlling for variation in economic conditions across regions and countries. Our findings show that fertility decline was strongly related to unemployment increase; this relationship was significant at different reproductive ages. Deteriorating economic conditions were associated with a stronger decline in fertility during the economic recession as compared with the pre-recession period. This evidence suggests the salience of factors such as broader perception of uncertainty that we could not capture in our models and which rose to prominence during the Great Recession. Furthermore, strongest fertility declines were observed in Southern Europe, Ireland and parts of Central and Eastern Europe, i.e. countries and regions where labour market conditions deteriorated most during the recession period. In Western Europe, and especially in the Nordic countries, fertility rates were not closely associated with the recession indicators.

**Electronic supplementary material:**

The online version of this article (10.1007/s10680-020-09556-y) contains supplementary material, which is available to authorized users.

## Introduction

The global *Great Recession* that started in autumn 2007 in the USA has hit almost all European countries, with many experiencing falling gross domestic product (GDP) and rising unemployment for most of the period of 2008–2013. The recession has destroyed many jobs, put a downward pressure on wages and induced a huge strain on government budgets, often resulting in spending cuts on social policies and families (OECD 2014). Previous economic recessions frequently led to fertility declines and stimulated fertility postponement (Sobotka et al. 2011; Cherlin et al. 2013). In particular, rising unemployment rates were associated with fertility declines that often took place with a time lag of one to two years (Simó Noguera et al. 2005; Berkowitz King 2005; Aaberge et al. 2005: 150; Adserà 2005,^2011^; Neels et al. 2013).

Most of the evidence on fertility changes in Europe after 2008 is in line with the past findings on pro-cyclical fertility. The increase in the period total fertility rates (TFRs) that started around the turn of the century has peaked in 2008–2010. Thereafter, fertility rates declined or levelled off in most European countries, especially among young women below age 25, accelerating the postponement of births to higher reproductive ages (Goldstein et al. 2013; Lanzieri 2013; Comolli 2017; Comolli et al. 2019). Altogether, 16 countries experienced a TFR decline by 0.1 or stronger between the year the TFR peaked in 2008–2011 and 2013. The fertility decline was more pronounced in countries and regions that experienced stronger economic downturns and faster increases in unemployment, especially in Southern Europe (Lanzieri 2013). However, fertility rates dropped also in the Nordic countries, which experienced rather mild economic decline (except in Denmark) and which continued providing extensive welfare and family policies (Comolli et al. 2019).

The majority of research on the consequences of the Great Recession on fertility in Europe has been descriptive (Lanzieri 2013) or confined to single countries (e.g. Pailhé and Régnier-Loilier 2015; Cazzola et al. 2016; Hilamo 2017; Kotzamanis et al., 2017). Few studies, which adopted a broader comparative perspective, demonstrated that an increase in unemployment during the economic recession was associated with declining fertility rates in Europe, especially at younger ages (Goldstein et al. 2013; Belido and Marcén 2016; Comolli 2017). These studies remained limited to country-level data, however.

Our study aims to expand the understanding of the links between economic conditions and fertility rates, focusing especially on the period of the Great Recession in Europe. Our main contribution is threefold. First, we investigate the links between changing economic and labour market conditions and fertility at sub-national level, providing a finer-grained contextual perspective than the research based on cross-country comparisons. Such approach allows us to capture changes in local employment and economic conditions in addition to country-specific conditions. It is also consistent with the previous research, which showed that both country and regional contexts can shape demographic and family processes, as they often differ in terms of jurisdiction, culture and economic conditions (Klüsener et al. 2013; Lappegård et al. 2017). The focus on smaller regions allows us also to account for sub-national diversity in the pre-recession economic conditions as well as the timing, severity and duration of the economic recession. Second, we explicitly focus on the fertility implications of changing economic and labour market dynamics, whereas most of the existing studies model the links between economic conditions and fertility. We are able to achieve these two objectives by employing a novel analytical approach—a three-level growth-curve model. It offers a flexible way of modelling change in the dependent variable after accounting for the interdependency of observations nested within the higher-level units (regions and countries). It thus allows us to investigate the links between labour market dynamics and fertility, while controlling for the variation in economic conditions across regions and countries. Finally, we also assess which changes in economic and employment conditions contributed most to the observed fertility decline during the recession.

## Economic Decline in Europe in 2008–2013

Deteriorating economic conditions are manifested by declining economic activity, as captured by a fall in the GDP, falling consumer confidence and adverse labour market trends. Worsening labour market conditions typically lead to stagnation or even decline of wages, higher incidence and persistence of unemployment and a spread of more uncertain employment forms. The labour market situation of young adults usually deteriorates most quickly as they tend to have lower qualifications, little work experience and poorly protected work contracts (Bruno et al. 2016). They are thus first to be laid off and last to be employed, which results in a rapid increase in youth unemployment (UN 2011). In addition, some young adults give up searching for a job altogether, expanding the ranks of those who neither work for pay nor participate in educational or training schemes (Scarpetta 2010), the so-called NEET (see, e.g. OECD 2016).

The economic recession, which started in 2008, affected the whole European continent. In its first phase, in 2008–2009, the economic output fell sharply in all parts of Europe. Subsequently, the economic trends varied between countries and regions. In some countries, a gradual economic recovery has taken hold since 2010, while other countries, especially in Southern Europe, experienced renewed economic downturn in 2010–2013. This second phase of the recession was partly fuelled by government austerity measures (House et al. 2017), which were motivated by declining tax incomes and rapidly rising levels of public debt.

The economic downturn was particularly severe in Southern Europe and in some countries in Central and Eastern Europe (CEE), especially in the Baltic countries, Bulgaria, Croatia or Hungary, where it was either very strong initially or lasted particularly long. Economic output also fell sharply in Ireland. In five Southern European countries (Cyprus, Italy, Greece, Portugal and Spain), GDP per capita contracted by over 10% in the period 2008–2013; in Greece the decline was as large as 32%. Economic contraction of more than 10% was also recorded in Ireland, Latvia, Croatia and Slovenia (Eurostat 2017). The falling economic output had severe repercussions for the labour market. Unemployment rates rose sharply, with the three Baltic countries (Latvia, Lithuania and Estonia), three Southern European countries (Cyprus, Greece and Spain) and Ireland experiencing double-digit increase in unemployment rate. Moreover, the recession also precipitated a sustained growth in long-term unemployment lasting 12 months or more.

Young adults below age 25 were hardest-hit by deteriorating labour market conditions. Their unemployment increased abruptly and even doubled or tripled in some of the countries, mainly in Southern Europe and in CEE (i.e. in the Baltic countries, Bulgaria, Croatia, Slovenia) and Ireland (OECD 2014 and 2016). The worsening situation of young adults is also reflected in an increase in the proportion of NEET. In Cyprus, Greece, Spain and Ireland, this proportion nearly doubled, but it also increased substantially in other Southern European and some of the CEE countries (Romania, Latvia, Croatia). The cuts in government expenditures disproportionally lowered spending on younger people and contributed to increases in poverty among children and young adults (OECD 2014: 112–113).

Only few European countries were relatively unscathed by the recession. Among them, Poland did not experience a single year of declining GDP in 2008–2013, three out of the five Nordic countries (Finland, Norway and Sweden), Belgium, France, Luxembourg and Malta saw only minor increases in unemployment and the three German-speaking countries (Austria, Germany and Switzerland) recorded very mild recession. Germany even displayed a decline in unemployment.

The dynamics of economic recession varied strongly not only between countries, but also at sub-national level. Several countries (UK, Germany, Bulgaria, Slovakia, Austria and Germany) had both regions which experienced overall a positive economic growth over 2008–2013 and regions where the economic output was shrinking heavily. For instance, in the UK the economy declined by more than 10% in two regions and at the same time it grew by around the same amount in North Eastern Scotland (Crescenzi et al. 2016). Youth unemployment changed in an even more diverse way: while it increased in most of the European regions, some countries experienced rising joblessness among the youth in some regions and declining in the others (e.g. Austria, Belgium, Czechia, France, Hungary, Norway, Sweden, UK and Slovakia). Regions differed not only with respect to the severity of the recession, but also in its timing and duration. For example, Northern Italian regions, which are more integrated with the global economy, were more strongly affected in the short term than Southern Italy where recession was milder at first, but was more protracted and its impact intensified with time (Crescenzi et al. 2016).

## Economic Downturn and Fertility: Past Research and Key Objectives of our Study

The economic decline directly affects many families and individuals by lowering their income, but also by fuelling general perceptions of uncertainty about future economic conditions (Kreyenfeld 2010; Kreyenfeld et al. 2012; Raymo and Shibata 2017; Vignoli et al. 2012, 2016; Ayllón 2019), even among those who are not directly affected by massive lay-offs or company bankruptcy (Sobotka et al. 2011; Hofmann et al. 2017). Consistently with the theoretical model developed by Ranjan (1999), young adults may avoid making long-term commitments—such as buying a flat, forming a union or having a child—in uncertain times. Instead, they may decide to live longer with their parents and prolong their education to increase their qualifications, escape unemployment and improve their position in the labour market (Kohler et al. 2002; Aassve et al. 2007). On the other hand, some couples may respond to higher economic uncertainty by having children, using temporary unemployment and lower wages as an opportunity window characterized by low opportunity costs of parenting, especially for the mothers (Butz and Ward 1979). In addition, women who lost their job may aim to have a child to improve their self-confidence and social status (Friedman et al. 1994).

All in all, the individual fertility responses to economic uncertainty are contingent on a wide range of circumstances and normative constraints and are likely to vary by age, sex and social status as well as by broader institutional conditions including welfare and family policies (Kreyenfeld 2010; Schmitt 2012; Vignoli et al. 2012, 2019). However, a vast body of research based on aggregate-level data shows that fertility is pro-cyclical and tends to fall when economic conditions deteriorate (Sobotka et al. 2011), suggesting that during the economic downturns most people tend to postpone childbearing rather than use the period of uncertainty to form a family. For instance, studies on the effects of the *Great Depression* of the 1930s demonstrated that a rapid surge in unemployment was followed by a drastic fall in fertility (Kiser and Whelpton 1953) and some of the births, presumably postponed during that era, have actually never taken place (Cherlin et al. 2013). Other studies, which cover the second half of the twentieth century, confirmed that young adults are more likely to postpone family formation during economic downturns (Neels et al. 2013; Hofmann et al. 2017) and even provide evidence for the negative effects of adverse labour market conditions experienced in young age for completed fertility (Currie and Schwandt 2014). The persistence of joblessness proved to be strongly negatively associated with fertility intentions (Busetta et al. 2019) and behaviour (Ciganda 2015), even in the aftermath of the Great Recession, when economic recovery was not immediately followed by creation of new jobs.

The effects of the recent economic recession on family formation have been widely studied in the USA. Schneider (2015) showed that deteriorating economic conditions and increasing uncertainty at a state level, manifested especially by rising unemployment and foreclosure rates, resulted in a decline of general fertility rate by 7.5%. Economic downturn contributed to an increase in childlessness by lowering first birth rates among women in their late thirties (Comolli and Bernardi 2015). Schneider and Hastings (2015) found that unemployment had the strongest negative impact on fertility of low-educated unmarried women. In addition, Seltzer’s (2019) research revealed a longer-term negative influence of labour market polarization, with deindustrialization and job displacement among middle-skilled workers fuelling sustained fertility declines that persisted after the economic recession ended.

Research for Europe was initially limited, but expanded in recent years. Some studies were descriptive (Lanzieri et al. 2013) or confined to single countries (Pailhé and Régnier-Loiler 2015; Cazolla et al. 2016; Hilamo 2017; Tragaki and Bagavos 2019). Other studies adopted a broader comparative perspective, mostly based on country-level data. Bellido and Marcén (2016) investigated changes in total fertility in response to an increase in unemployment using a pooled sample of 30 European countries over the period 1991–2013. Goldstein et al. (2013) looked, in addition, at changes in age-specific fertility rates by birth order, but covered only the first stage of the economic recession until 2010. Comolli (2017) extended the study by Goldstein et al. (2013) using longer time series and a wider selection of economic indicators. These studies demonstrated that an increase in unemployment during the economic recession was associated with declining fertility rates in Europe, especially at younger ages. In addition, Comolli (2017) showed that fertility responded negatively to changes in consumer confidence. A detailed study of the Nordic countries by Comolli et al. (2019) revealed a uniform negative fertility response to the economic recession across countries and education groups, which contrasted with the more differentiated fertility developments by social group in the previous periods of economic downturns. Ayllón’s (2019) research, combining individual-level data with regional (NUTS-1) indicators of unemployment, precarious employment and subjective indicators of job uncertainty, is to our knowledge the only recent European comparative study on fertility change that analysed sub-national data. Objective indicators of job insecurity, in particular unemployment and long-term unemployment, were more strongly linked to fertility than subjective indicators of job perceptions, which varied by context.

Previous studies focused also on investigating the links between economic conditions and fertility (e.g. Schneider 2015; Ayllón 2019; Seltzer 2019). This “static” design may distort the perceptions of the effects of economic changes on fertility, as the reported effects may result from the long-term differences between countries and regions as well as from the economic dynamics over time. As such, they blend two important and distinct questions, “Do economically worse off regions have lower fertility rates?” and “What is the impact of deteriorating economic conditions on fertility?”, together. Equally theoretically important is the question whether fertility responses to changing economic conditions are nonlinear and generally stronger during the recession period, possibly due to rising anxiety, uncertainty and negative expectations, even among the people not directly affected by the recession (Sobotka et al. 2011).

Our study thus builds upon previous research, aiming to expand the understanding of the links between economic conditions and fertility in several directions and taking into account the shortcomings of previous research. In particular, we aimed to (i) better account for both the local conditions and the country context than most of the past studies which focused on a national level only; (ii) focus on the fertility consequences of changing economic and labour market dynamics rather than simply studying the links between economic conditions and fertility; (iii) investigate whether fertility responses to changing economic conditions strengthened during the recession period; and (iv) assess which changes in economic and employment conditions contributed most to the observed fertility decline during the recession. To this end, we implement a three-level growth curve model, which allows us to account for national and sub-national context and separate the effects of changes in economic conditions on fertility from the cross-regional associations between the two variables.

## Data and Indicators

Our selection of data and indicators aimed to provide a wide coverage of European countries and sub-national regions to capture the effects of the changes in local economic conditions on fertility. We used data for NUTS-2 regions, which are roughly equally populated (population 0.8–3 million), and they are the smallest units for which we could access comparable and reliable data on fertility and economic conditions for the analysed countries. As of January 2017, there were 276 NUTS-2 regions in the 28 EU countries including United Kingdom.

To measure fertility, we used the TFR, complemented by its age-specific components—cumulative age-specific fertility rates (ASFR) computed for the age groups 15–19, 20–24, 25–29, 30–34 and 35+. These data, published by Eurostat at the NUTS-2 level since 1999 onward, are computed by combining national vital statistics on births by age of mother and official data and estimates on female population by age. While birth registration is complete or almost complete in Europe, the fertility rates may be underestimated and the fertility time series can be distorted by series breaks and jumps in some countries due to incomplete registration of migration (especially outmigration) and inconsistent and changing rules on the registration of births to women who left the country. These problems are most pertinent in the countries of Central and Eastern Europe with sizeable outmigration (especially Bulgaria, Romania, Poland, Hungary, Slovakia and the three Baltic countries), therefore, our findings for these countries should be interpreted with caution.

To measure the changing economic and labour market conditions, we use economic and labour market indicators, which closely reflect the economic cycle and which indeed deteriorated during the Great Recession. The selection of our explanatory variables was limited by data availability and reliability. First, we considered a range of labour market measures which indicate the instability of employment and persistence of joblessness, such as unemployment rate (overall unemployment and youth unemployment at ages below 25) and the proportion in long-term unemployment (unemployment lasting 12 months or more). These indicators originate from the European Labour Force Survey (ELFS), which is a comparative large-sample survey designed for collecting labour market data of high quality. These data are available in Eurostat Database as annual aggregate indicators provided for the NUTS-2 units for the period since 1999. Unfortunately, the database did not provide data on temporary employment at the NUTS-2 level.

Second, we accounted for the proportion of young adults (aged 18–24) not in employment, education or training (NEET), also collected in ELFS. The NEET indicator was criticized in the literature for being heterogeneous and consisting not only of the unemployed and disengaged, but also young care providers, persons with health problems and disabilities as well as persons who are actively involved in artistic activities or self-directed training (Cavalca 2016). In Northern and Western Europe, the NEETs are mostly the short-term unemployed, in Southern Europe this group includes mainly the long-term unemployed and discouraged workers and in CEE countries it includes many women with family responsibilities (Eurofound 2016). Despite this diversity, the NEET indicator is an essential tool for a better understanding of the vulnerability of youth in terms of their labour market participation and risk of social exclusion (Eurofound 2016). In our view, an increase in the share of NEETs, observed during the economic recession, suggests an increase in youth disengagement during that time. Furthermore, the high share of NEET population signals an exposure to informal work, insecure and low wage employment in future (Furlong 2006). Finally, we included the annual rate of GDP decline as it can be considered as a proxy of the general economic prosperity and economic trends. To this end, we used data on GDP at current prices in EUR, which is available in Eurostat since 2000. We converted it into local currencies using the average annual exchange rates and adjusted it for inflation using the harmonized index of consumer prices. Next, we computed relative annual regional GDP growth rates for the years 2001–2014.^1^

We lagged the economic and labour market indicators by one year.^2^ Our dataset therefore consists of the series of labour market and economic indicators for the years 2001–2013 and the series of fertility indicators for the period 2002–2014. Out of the 276 NUTS-2 regions in the EU-28 countries, we excluded ten overseas territories, which often differ widely in their cultural and institutional characteristics from the European regions. Further, we excluded data for another 16 regions and used the data for the whole Slovenia instead of its two NUTS-2 regions. These regions were excluded due to boundary changes or missing labour market indicators (see Appendix 1 for the list of omitted regions).

Overall, our sample comprised 251 NUTS-2 regions, nested within 28 countries. In this dataset, fewer than 5% of observations per variable are missing and fewer than 8% are classified by Eurostat as low reliability values. However, variables based on small populations, i.e. the youth unemployment rate and long-term unemployment, contain more missing values (10–11% of all observations) and low reliability values (22–24%) (for details see Appendix 2). In order to minimize the number of missing values, we imputed the data with a cubic spline interpolation. We performed this imputation only if there were no more than four missing values in a row and if they were part of the time series, preceded and followed by known values.

All indicators we considered show a sharp deterioration in economic conditions after 2007, especially in 2009. According to the conventional indicator of GDP growth, the economic recession was over by 2013, with more than half of the regions registering positive GDP change. However, the labour market indicators such as unemployment rate and long-term unemployment still increased in most regions in that year. Finally, the TFR trends show a reversal of fertility across most regions after 2007, but at the same time fertility rates did not become more differentiated across regions.

## Model

Our data have a hierarchical structure. Year-observation units are nested within regions and regions are nested within countries. Using these data, we estimated three-level growth-curve models (GCMs) with regional TFR and ASFRs as dependent variables. The GCM offers a flexible way of modelling change in the dependent variable after accounting for the interdependency of observations nested within higher-level units (Rabe-Hesketh and Skrondal 2012). The functional form of the pattern of change in the dependent variable and the parameters describing the change (intercepts and slopes) can be set to vary across the studied subjects. Furthermore, the GCM allows for investigating the effects of the subjects’ characteristics (both time-constant and time-varying) on the change in the dependent variable and the effects of the time-varying characteristics can be set to vary across subjects. This means that using the GCM we can model the association between the change in regional economic conditions and the developments in regional fertility by allowing the trend parameters to vary across regions or countries and accounting for the dependency of the observations within NUTS-2 regions and countries. We can also investigate how the relationship between economic conditions and fertility varies across higher-level units, in our case NUTS-2 regions or countries.

We used the mixed-effect approach to growth-curve modelling which fits the GCM within a regression framework. This approach is better suited to modelling growth in one observed outcome variable, time-unbalanced data and models with more than two nesting levels than the alternative latent curve approach, usually fitted with structural equation models (Steele 2008; McNeish and Matta 2018). Our GCM originates from the following simple specification:

1 $${\text{FR}}_{\text{crt}} = \beta_{0} + \mathop \sum \limits_{j = 1}^{m} \beta_{j} \cdot t + \mathop \sum \limits_{i = 1}^{n} \gamma_{i} \cdot X_{icrt - 1} + \varepsilon_{\text{crt}} \quad i = 1,\,2,\,3,\,4,\quad j = 1,\,2,\,3$$

where ${\text{FR}}_{\text{crt}}$ denotes TFR or cumulated ASFRs in country *c*, region *r* at time *t*; $\beta_{0}$ stands for the overall intercept; and $\beta_{j}$ are the slopes in the piecewise linear spline which is used for modelling the time trend in fertility in *j* a priori defined intervals. We set the nodes of the piecewise linear splines at the end of 2008 and 2010, i.e. the time points when we observed a change in fertility trend in most of the analysed countries. The $X_{icrt - 1}$ denotes the economic or labour market indicator *i* measured in country *c*, region *r* at the time *t*-1, i.e. lagged by one year. In all models, $X_{icrt - 1}$ represent the unemployment rate, proportion of long-term unemployed, the share of NEET as well as the GDP growth. The correlations between these economic indicators do not exceed 0.7 (and usually are smaller than 0.5), which allowed us to introduce all economic indicators into one model without risking multicollinearity problems. We also confirmed that multicollinearity is not a problem by computing the Variance Inflation Factors (VIF), which appeared to be lower than 2.5 on most of our major explanatory covariates (see Appendix 1 in the online supplementary material). The unemployment rate at ages 20–64 enters the model for the TFR, the youth unemployment rate (ages 15–24) is included in the models for ASFR at ages 15–19 and 20–24 and the unemployment rate of persons aged 25–64 is included in the models for ASFR at ages 25–29, 30–34 and 35+. All economic indicators are centred at the sample grand mean to facilitate the interpretation of our findings.

The model in Eq. 1 informs us how the overall variation in economic conditions (within regions, between regions within each country and between countries) is correlated with the overall variation in fertility rates, but does not allow us answering the question how the within-region change in economic conditions affects the change in regional fertility. In order to provide the answer to the latter question, we decomposed the variation in a variable $X_{icrt - 1}$ into the within-region variation $\left( {X_{icrt - 1} - \overline{{X_{icr \cdot} }} } \right)$, between-region within-country variation $\left( {\overline{{X_{icr \cdot} }} - \overline{{X_{ic \cdot \cdot} }} } \right)$ and between-country variation $\overline{{X_{ic \cdot} }}$:

2 $$X_{icrt - 1} = \left( {X_{icrt - 1} - \overline{{X_{icr \cdot} }} } \right) + \left( {\overline{{X_{icr \cdot} }} - \overline{{X_{ic\cdot \cdot} }} } \right) + \overline{{X_{ic \cdot \cdot} }}$$

where $\overline{{X_{icr \cdot} }}$ denotes the average level of variable *X*_*i*_ in a country *c* and region *r* (regardless of time), and $\overline{{X_{ic \cdot \cdot} }}$ stands for the average level of variable *X*_*i*_ in a country *c*. Furthermore, the model in Eq. (1) does not yet account for the country- and region-specific variation in initial fertility levels, trend parameters and fertility responses to changes in economic conditions. Implementing Eq. (2) into (1) and accounting for country-/region-specific random effects gives the following model specification:

3 $$\begin{aligned} {\text{FR}}_{crt} & = (\beta_{0} + \mu_{0c} + \mu_{0r} ) + \mathop \sum \limits_{j = 1}^{m} \left( {\beta_{j} + \mu_{jc} } \right) \cdot t \\ & \quad + \mathop \sum \limits_{i = 1}^{n} \left[ {(\gamma^{\text{WR}}_{i} + \mu_{ic} } \right) \cdot \left( {X_{icrt - 1} - \overline{{X_{icr \cdot} }} } \right) + \gamma^{\text{BRWC}}_{i} \left( {\overline{{X_{icr \cdot} }} - \overline{{X_{ic \cdot \cdot} }} } \right) + \gamma^{\text{BC}}_{i} \overline{{X_{ic \cdot \cdot} }} ] \\ & \quad + \varepsilon_{crt} \\ \end{aligned}$$

$\gamma^{WR}_{i}$ constitutes the main coefficient of our interest. It denotes the within-region effect of economic conditions on fertility and tells us by how much fertility increased/declined as a result of a temporal change in an economic indicator. The remaining two coefficients, $\gamma^{\text{BRWC}}_{i}$ and $\gamma^{\text{BC}}_{i}$, inform us about how the between-region within-country and between-country variation in economic conditions relate to the within-country and between-country variation in fertility rates. They thus denote associations between economic conditions and fertility within the country and between countries and constitute control variables for our main variables of interest.

Country- and region-specific idiosyncrasies are modelled with a series of random terms. The random intercepts $\mu_{0c} {\text{ and }} \mu_{0r}$ represent country- and region-specific deviations from the overall intercept $\beta_{0}$ and thus allow for a within- and between-country variation in the initial fertility. The random slopes $\mu_{jc}$ and $\mu_{ic}$ represent country-specific shifts in the spline slopes $\beta_{j}$ and the within-region effects of economic conditions $\gamma^{WR}_{i}$, respectively, and allow for the cross-country variation in fertility developments over time and cross-country variation in fertility responses to changes in economic conditions. The covariances between the random effects turned out to be insignificant and thus were set to zero.^3^ We decided to introduce the random slopes at the country level instead of the NUTS-2 level to be able to conclude how the regional effects aggregate into country-level effects.

Finally, $\varepsilon_{crt}$ constitutes the level-1 residual. We allowed for the serial autocorrelation of the level-1 residuals. The order of the autoregressive process was selected for each model separately: the residuals in the model for the TFR required an introduction of the AR(3), which means that error terms three periods away from each other were correlated. Models for the age-specific fertility rates required the AR(2), although for modelling fertility at ages 20–24 and 25–29 we had to use the AR(1) as the models using higher-order correlation faced convergence problems. The model expressed in Eq. (3) is our final model. As it is quite complex, we have also specified more parsimonious models, e.g. random intercept models with and without a decomposition into within-region, between-region within country and between country variation, or a fixed-effects model. These models do not account for the hierarchical structure of our data (fixed-effects model), country-specific variation in fertility responses to changes in economic conditions (all alternative specifications) or country- and region-specific unobserved time-invariant characteristics (random-intercept models without a decomposition). Indeed, they display a weaker fit to the data (see Appendix 2 in the online supplementary material) and thus were rejected in favour of the model in the Eq. (3). 

## Empirical Findings

Our model provides estimates of within-region $(\gamma_{i}^{\text{WR}} )$ as well as between-region within-country effects $(\gamma_{i}^{\text{BRWC}} )$ of economic conditions on fertility. The results confirm that both between-region within-country differences as well as changes in economic conditions over time are related to fertility. To keep our study focused, however, we do not discuss the correlations between the economic conditions and fertility across regions. Instead, we focus on the estimates of the association between the change in economic conditions and changes in fertility, which address our research objectives. To provide a comparable perspective on the effects of individual components of economic and labour market changes, we present average annual changes in fertility rates, which would be observed in a hypothetical situation if unemployment rate, share of long-term unemployed and the share of NEET increased by 10 percentage points (pp.) and the annual rate of GDP growth declined by 10 pp. Finally, to obtain a more realistic picture of fertility responses to changing economic conditions, we predict total fertility in 2009–2014 under five scenarios. The discussed findings are displayed graphically, while the full model estimates are presented in Appendixes 3 and 4.

### Changes in economic conditions and fertility dynamics

**Fig. 1:**
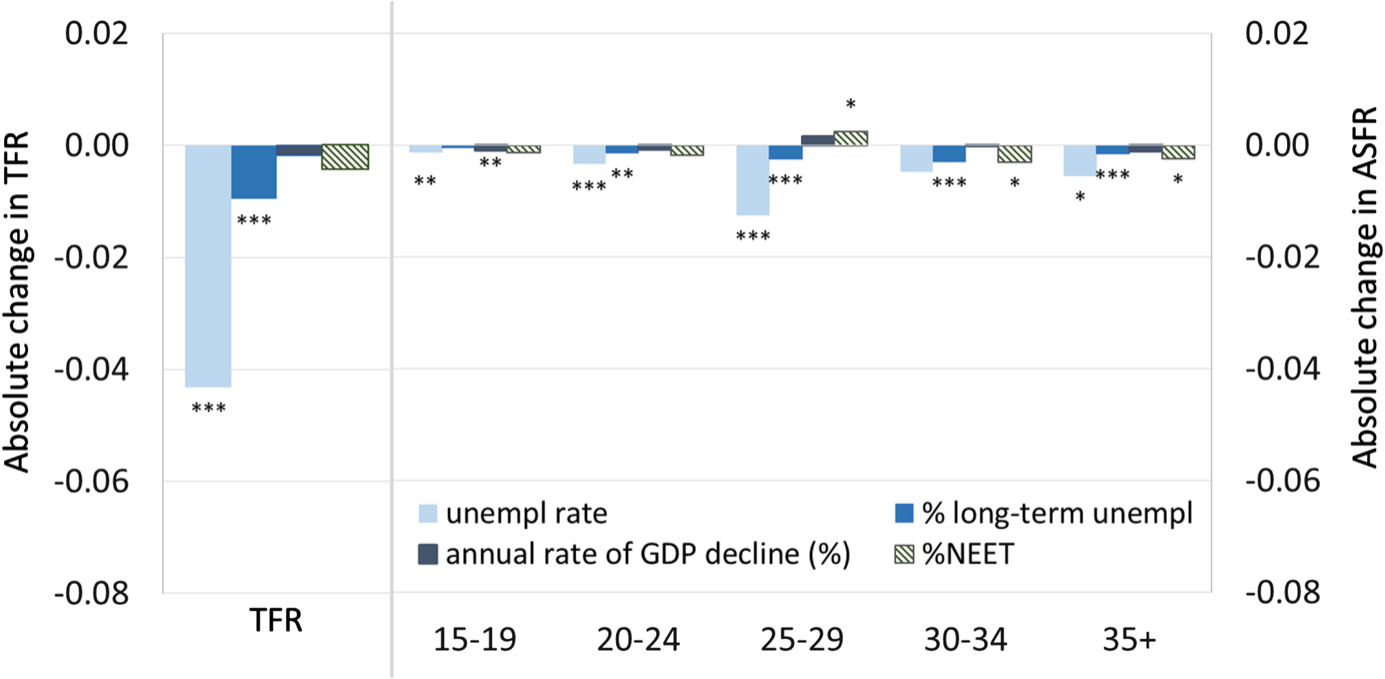
Absolute change in total fertility rate (left panel) and cumulated age-specific fertility rates (right panel) resulting from the 10 pp. increase in unemployment rate, % long-term unemployment and % NEET and 10 pp decline in GDP growth. NUTS-2 regions in EU-28, 2001–2014. Note: Unemployment rate in the model for total fertility refers to the age group 20–64, in the models for fertility at ages 15–19 and 20–24 to the age group 15–24 and in the models for fertility at ages 25+ to the age group 25–64. *P* value: *−  0.1, **−  0.05, ***−  0.01

The deterioration of economic and labour market conditions was related to fertility decline across all reproductive ages, including higher reproductive ages when further postponement of fertility may limit the chances for future pregnancy (Fig. 1). Fertility decline was most strongly associated with an increase in unemployment. Our model, as specified in Eq. (3), predicted that a hypothetical increase in unemployment rate by 10 percentage points (pp.) would lead, on average, to a decline in total fertility by slightly more than 0.04, net of all other covariates we considered. The negative relationship between unemployment and fertility was most significant at younger age groups (below age 30).

An increase in the proportion of long-term unemployed was also significantly related to fertility decline at all ages except for the youngest age group (15–19). Fertility response to an increase in long-term unemployment was, however, much weaker than to an increase in overall unemployment rate.

Changes in the remaining two economic indicators—the rate of GDP decline and the share of NEETs—were only weakly related to changes in fertility when considered jointly with unemployment rate and long-term unemployment. Stepwise analyses showed that the GDP decline and the change in the share of NEETs were losing their association with fertility after we accounted for unemployment and long-term unemployment. This suggests that worsening of economic conditions is related to fertility mainly through employment-related conditions.

### Fertility Responses to Changing Economic Conditions Before and During the Great Recession

**Fig. 2:**
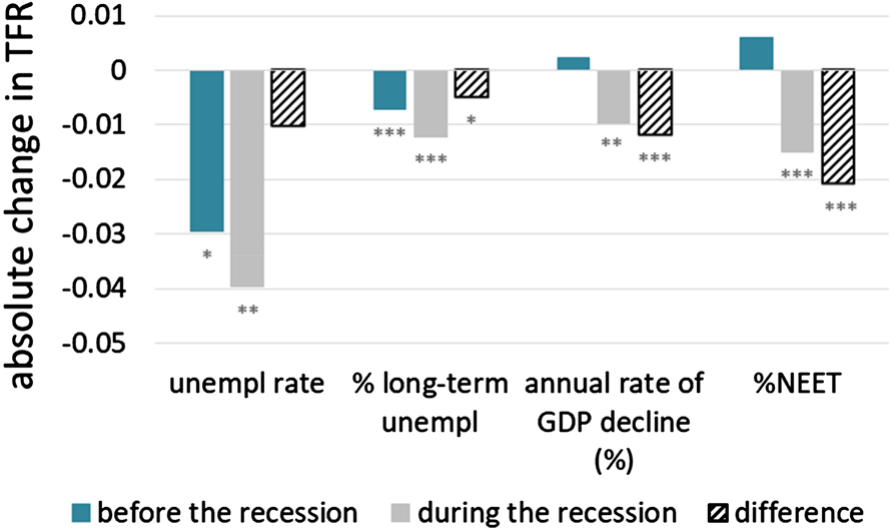
Absolute change in total fertility rate resulting from the 10 pp. increase in unemployment rate, % long-term unemployment and % NEET and 10 pp. decline in GDP growth within NUTS-2 regions in two periods: before (2001–2008) and during (2009–2014) the economic recession. Note: see Fig. 1

In order to evaluate whether the correlation between changing economic conditions and fertility was stronger during than before the recession, we interacted the within-region variation in economic conditions $\left( {X_{icrt - 1} - \overline{{X_{icr} }} } \right)$ with the recession dummy, set at 1 in the period 2008–2014 and 0 otherwise. Our findings largely support this hypothesis: deteriorating economic conditions were associated with a stronger decline in total fertility during the economic recession as compared with the pre-recession period (Fig. 2). The decline in the GDP and an increase in the share of NEETs, which were not related to changes in the TFR before the recession, turned out to depress it after 2007. Furthermore, the negative association between changes in long-term unemployment and total fertility became slightly stronger. The negative relationship between unemployment and the TFR remained equally strong before and during the recession.

Why are the fertility reactions to changing economic and labour market conditions stronger during the economic recession than before? Is it because the relationship between economic conditions and fertility is nonlinear? For instance, Garfinkel et al. (2016) found that Americans react more strongly to deterioration in economic conditions than to their improvement. Furthermore, it is also possible that a worsening in economic conditions is particularly detrimental for fertility at high levels of unemployment or low levels of economic growth (or economic decline). We excluded these possibilities by interacting the within-region variation in economic conditions $\left( {X_{icrt - 1} - \overline{{X_{icr} }} } \right)$ with (a) a dummy denoting whether the economic indicator *i* was increasing (as opposed to remaining stable or declining)^4^ (b) a dummy denoting whether the economic indicator was exceptionally high, e.g. above the regional average (versus staying below or at the regional average) (see the results in Appendix 4). We found no support for the hypothesis that fertility reacts more strongly to a worsening rather than an improvement in economic conditions. Neither did we find evidence that economic downturn is more strongly related to fertility decline when the initial conditions are poor than when they are good. Overall, our findings suggest that economic conditions had a stronger impact on fertility after 2008 than before due to an accumulation of negative economic developments (some of which may not have been captured by our model) and, we reckon, a general increase in uncertainty about the future. We elaborate on this in the concluding discussion.

### The Links Between Changing Economic Conditions and Fertility Dynamics by Country

**Fig. 3:**
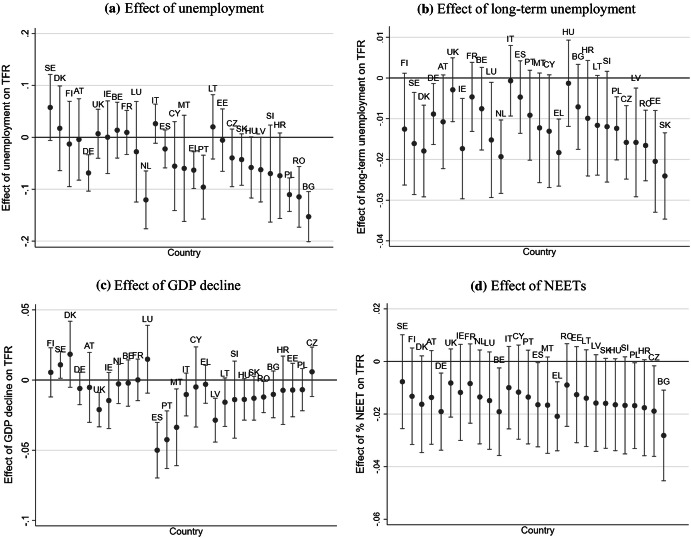
Absolute change in TFR resulting from the 10 pp. change in economic conditions by country, 2009–2014. 90% CI

Our empirical strategy allows us also to investigate variation in post-2008 fertility effects by country. To this end, we computed the empirical Bayes predictions of the random slopes $\mu_{ic}$ and their variances. We obtained the country-specific fertility effects by summing the average within-region effect $\gamma_{i}^{WR}$ with the predicted country-specific parameters $\mu_{ic}$. The variance of the country-specific effects was obtained in a similar way. Our findings are presented in Fig. 3a–d. Many of the obtained effects are only marginally significant as the number of countries in our study is relatively small. Despite this shortcoming, our findings are consistent with analyses we performed on country groups (Matysiak et al. 2018), except for the CEE countries for which this study revealed a substantial heterogeneity. Most of the predicted associations are negative, and, consistent with our findings discussed above, unemployment and long-term unemployment play the strongest role. Furthermore, fertility effects of changing economic conditions are strongest (and often significant) in countries which were most strongly hit by the economic recession, especially in Southern Europe (Greece, Portugal, Spain, Malta) and some of the Central and Eastern European countries (Romania, Bulgaria, Czechia, Slovakia, Latvia, Estonia). Declining GDP, which displayed no significant correlation with fertility on average, clearly contributed to fertility decline in some of the Southern European countries (Greece, Malta, Portugal), CEE countries (Latvia and Romania) and in the UK. Finally, significant fertility reactions are identified in the Netherlands and Germany. The latter was only weakly affected by the economic crisis and unemployment there was declining during the recession period.

### Fertility responses to the actual changes in economic conditions

So far, our analysis has focused on discussing how fertility changes were associated, on average, with a hypothetical 10 pp. change in each of the indicators of economic conditions we considered. Such hypothetical changes often differ widely from the actual dynamics in economic and employment conditions in individual regions. To obtain a more realistic picture of fertility responses to changing economic conditions, we predict total fertility in 2009–2014 under five scenarios, out of which the first four are hypothetical. To this end, we use our model presented in Eq. (3) with an additional interaction between the within-region variation in economic conditions $\left( {X_{icrt - 1} - \overline{{X_{icr} }} } \right)$ and the recession dummy, which proved to be essential. The first scenario, “no-recession scenario” (S1), predicts TFR as if there was no recession and economic conditions remained constant at 2007 level (prior to the economic recession). Subsequently, we predict the TFR allowing for actual changes in unemployment (S2), unemployment and long-term unemployment (S3), and unemployment, long-term unemployment and the GDP (S4). Finally, the “Full model” (S5) predicts the change in total fertility based on the observed changes in the complete set of indicators. The latter scenario was computed not only for the recession period, but also for the pre-recession years. This strategy allows us to compare the observed TFR with the TFR predicted by our model and verify in this way its validity.

In the first step, we compare the actual TFR with the TFR predicted under S5 (in which all economic indicators assume their actual values). It turns out that our model predicts the actual TFR very well. The absolute difference between the predicted and actual TFR does not exceed 0.05 in 55% of cases and is smaller than 0.1 for 84% of observations in our sample. The distribution of the average regional absolute differences (Appendix 5) reveals that large differences are not geographically clustered, but are evenly distributed across the continent.

**Fig. 4:**
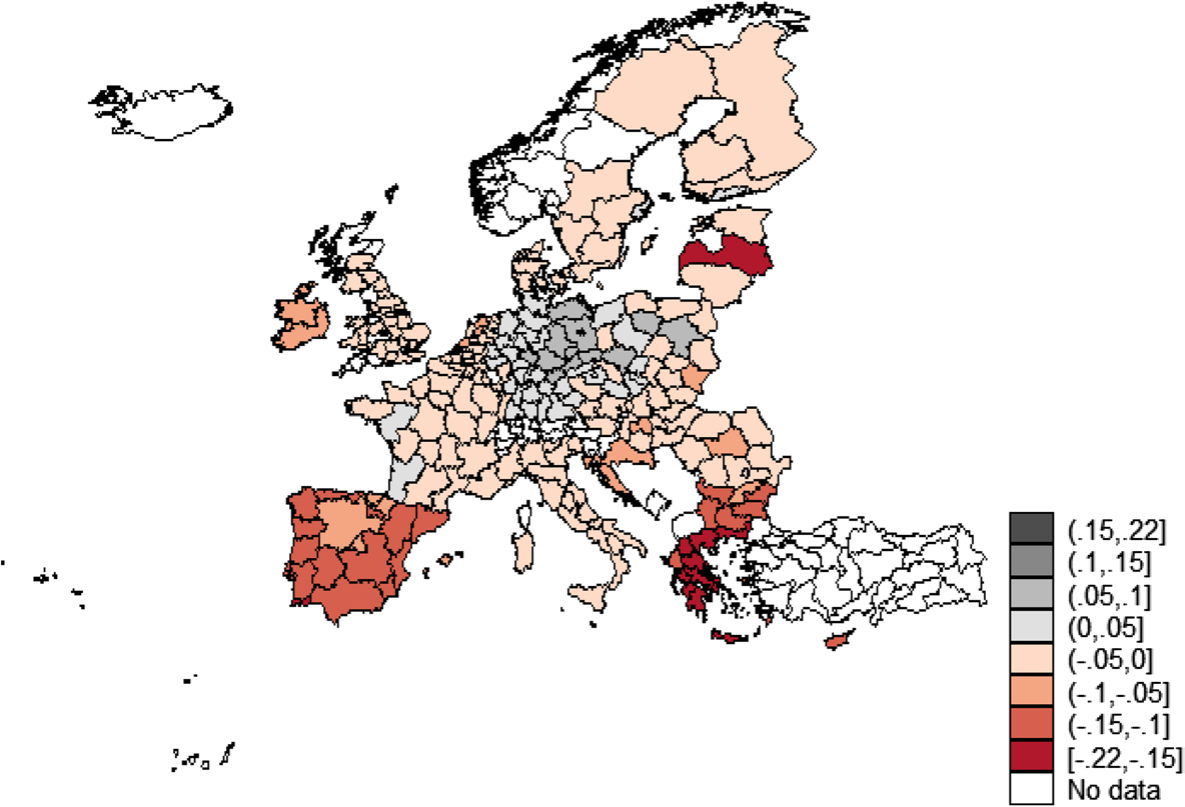
Predicted changes in the TFR due to the economic recession, 2009–2014, NUTS-2 regions of the EU-28 including UK

Next, we map the maximum difference between predictions from scenarios S1 (no recession) and S5 (predicted TFR given the actual economic conditions) for all analysed regions in 2009–2014 (Fig. 4). This comparison allows us to assess the maximum fertility effects of the actual change in economic indicators considered in our model. For most regions, the difference was largest during the last two years analysed, 2013 and 2014. Regions in the UK and in the Baltic countries, where economic recession was strong but relatively short, as well as Finnish, Swedish and some of the Polish and Romanian regions experienced the strongest fertility effects in earlier years. The shades of red, observed in most of the regions, demonstrate the maximum decline in fertility, resulting from a worsening in economic conditions, while the shades of grey display the maximum increases in fertility, resulting from an improvement in economic conditions. The latter are displayed for regions where no fertility decline took place during the recession. These regions form a large cluster in Central Europe, encompassing most of German and Polish regions and several regions in Belgium, Czechia as well as the Vienna region in Austria. This should not come as a surprise, as Poland did not experience economic decline around 2008–2010 and most other countries in the region saw relatively mild economic recession. The recession-related fertility decline was strongest in Southern Europe. The total fertility would be by 0.15 or more higher in most of the Greek regions and by 0.10–0.15 higher in most of the Spanish and Portuguese regions if economic conditions had not changed after 2007. Substantial fertility decline due to economic recession was also recorded in Ireland and in some of the Eastern European regions, especially in Latvia (by 0.22) and in most of the Bulgarian and Croatian regions. In other parts of Europe, recession-induced fertility decline was, however, small if it took place at all.

Most countries experienced relatively little regional variation in recession-induced fertility declines. However, there were notable regional differences within and across countries, including larger fertility increases in eastern Germany as compared with western Germany and an East–West gradient in fertility change in Poland (with fertility increases due to positive economic conditions mostly concentrated in the western part of the country). In addition, there were relatively large regional differences in recession-related fertility declines in several strongly affected countries, especially Greece, where the recession-induced TFR change ranged from − 0.11 in Ionian Islands to − 0.20 in Crete.

**Fig. 5:**
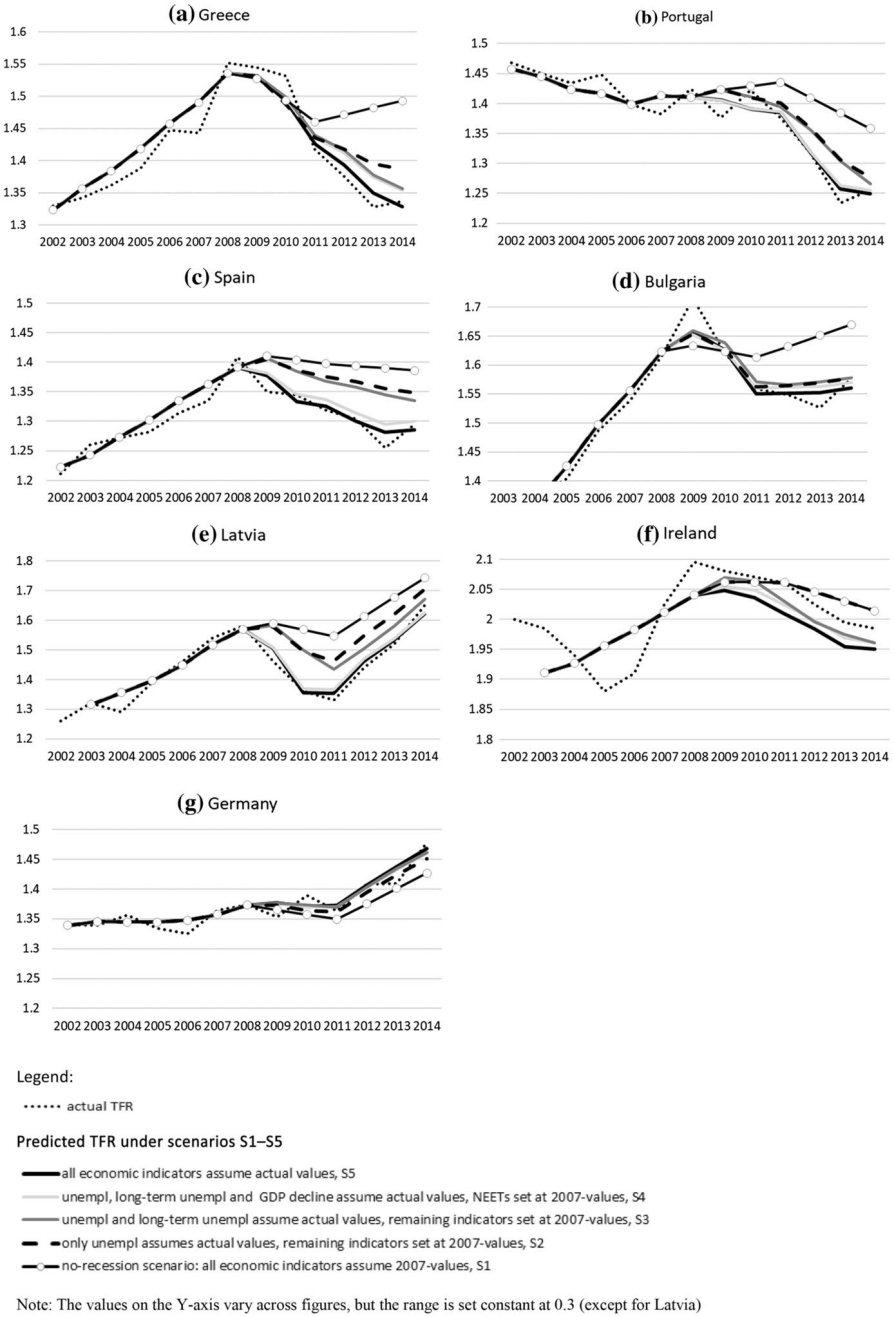
Observed and predicted total fertility rate under scenarios S1–S5 by country, 2002–2014

Finally, we investigated which economic factors contributed most to fertility decline during the recession. To this end, we compared total fertility predicted under the no-recession scenario to the total fertility predicted under scenarios S2–S5 (i.e. after allowing stepwise for actual changes in unemployment, long-term unemployment, GDP decline and NEETs). We present our findings in Fig. 5a–g for countries, where fertility effects were the strongest—Greece, Spain, Portugal, Latvia, Bulgaria and Ireland. In addition, we also display fertility developments in Germany, where fertility increased during the recession period and unemployment declined. We do not present the country-level predictions for the remaining countries, because fertility reactions to the changes in economic conditions are so small there that the predicted fertility rates from scenarios S1–S5 largely overlap (these figures are available upon request).

Our findings suggest that fertility decline was associated with an increase in unemployment and in Southern Europe and Latvia, in addition, with economic decline as measured by falling GDP. A different pattern was observed only in Ireland, where fertility decline was largely associated with an increase in long-term unemployment. In Germany, where an increase in fertility was recorded, it was achieved mainly in association with falling unemployment rates during the recession.

### Robustness checks

Our findings have passed several robustness checks. First, we verified whether lagging our economic indicators by one year is sufficient to capture fertility reactions to changing economic conditions. To this end, we introduced a two-year lag (*t-2*) in economic indicators in addition to the one-year lag (*t-1*) incorporated in our model. Results are displayed in Appendix 3 in the online supplementary material. Even though changes in some of the economic indicators at *t*-2 turned out to be significantly related to fertility (this was mainly the case of unemployment rate), introducing them into the model did not change the significance or the direction of the estimated coefficients at* t-1* in the vast majority of cases.^5^ Furthermore, the BIC statistic clearly showed that the more parsimonious models, with lags at *t*-1 only, displayed a better fit.

Second, we verified whether the imputation of missing values could have affected our findings. To this end, we re-estimated our models using the pre-imputation dataset. Hardly any changes have been observed in our findings (see Appendix 4 in the online supplementary material).

## Concluding discussion

The *Great Recession* had a long-lasting impact on income, employment, health, well-being and family behaviour of Europeans. Our main focus was on investigating how economic and labour market dynamics relate to fertility dynamics while controlling for the variation in economic conditions between regions in each country and between countries. We covered a range of economic and labour market indicators to capture the multifaceted configuration of the economic recession and explore huge sub-national variation in its progression. To that aim, we have incorporated long-term unemployment and the share of young adults who are not in education, employment or training. Our models, including the full set of indicators, provided a close fit to the actual fertility trends in most of the sub-national regions in Europe.

Three general findings are consistent across wider groups of countries and are also in line with the past findings reported at a national level. First, the periods of economic downturns are associated with negative fertility dynamics across the countries and regions analysed. Second, the role of unemployment and long-term unemployment clearly dominates over the other economic indicators studied, which suggests that worsening economic conditions relate to fertility mainly through deteriorating labour market. In particular, changes in unemployment rate showed the strongest link to fertility dynamics across all ages: an increase in unemployment rate by 10 pp was associated, on average, with a decline in total fertility by nearly 0.04 in absolute terms. In addition to unemployment, GDP decline was closely associated with fertility decline in Southern Europe and Latvia. Third, fertility rates were negatively linked with deteriorating economic conditions across the whole range of childbearing ages, including late reproductive ages when women who postpone childbearing may not be able to realize their fertility plans later in life. The latter finding suggests that economic recession might have affected not only the timing of fertility, but also its quantum.

We were able to provide a finer picture of the factors associated with fertility dynamics in individual countries and sub-national regions in Europe. Our results suggest that country context played a dominant role in recession-related fertility declines, as fertility change was strongly differentiated by country and in most countries, the regional variation in recession-related fertility change was relatively low. This is in line with large between-country differences in the severity of the economic recession and policy responses to it. Yet, there was notable regional variation in several countries, especially in Greece, as well as broader clusters of similar recession-induced fertility changes (or the lack of them) spanning across country boundaries, especially in Central Europe, where the recession was mild. Our model, which controls simultaneously for sub-national and country effects (regions are nested within countries) and accounts at the same time for the levels and changes in economic/labour market conditions, arguably constitutes an improvement to the models used in the past. We show that across Europe, both between-country and within-country variations in economic conditions are relevant for understanding fertility dynamics. The between-country dimension proved to be especially relevant, in agreement with the results of other recent studies (Klüsener et al. 2013; Lappegård et al. 2017; Fox et al. 2019). In particular, our findings are most informative for the countries and regions that experienced most severe economic setbacks, i.e. Southern Europe as well as in parts of Central and Eastern Europe. In the regions of Greece, Spain and Portugal, deteriorating economic and labour market conditions affected the period TFR by − 0.08 (Basque country in Spain) to − 0.20 (Crete in Greece) between 2007 and 2014 compared with the “no-change” scenario. This decline was largely attributable to an increase in unemployment and GDP decline. These factors, especially unemployment and long-term unemployment, also played a dominant role in fertility decline in Central and Eastern Europe.

The negative effects of GDP decline and of the rising share of NEETs as well as long-term unemployment were more pronounced during the recession period in 2008–2014. This strengthening of the negative influence of economic conditions on fertility during the recession did not result from the fact that Europeans adjust their fertility more strongly to the worsening rather than improvement of economic conditions (as it has been found for the USA). Moreover, in Central Europe and in the Nordic countries fertility rates were not closely associated with the recession indicators. This suggests that our indicators have tapped into the factors we could not capture in our models and which rose to prominence during the recession. These include rising poverty and economic deprivation, lower income, and also wider structural changes in the labour market characterized by falling opportunities for the middle-income workers and the spread of lower-paid temporary contracts (Adsera 2017; Seltzer 2019). Our findings may also reflect a specific period effect when economic uncertainty diffused across Europe, fuelled by negative expectations and declining confidence about the future, not captured in our data. Sobotka et al. (2011) emphasized the role of expectations regarding future negative economic trends that may result in feelings of anxiety and depression, in shaping fertility. In this context, individuals’ perceptions of the broader economic climate, including media coverage of the economy, might increase uncertainty and affect fertility (Vignoli et al. 2020). We hope our results will stimulate future research in which this hypothesis is explicitly tested.

Our study has limitations. First, it is difficult to evaluate whether the observed fertility declines were mostly driven by the temporary postponement of childbearing during uncertain times or rather by a fall in the underlying level (quantum) of fertility that will also depress completed family size of women who were in prime reproductive ages around 2010. Even though the declines in fertility of women aged 35+ suggest some lasting influence on fertility levels among women approaching the end of their reproductive lives, these effects are rather small. Second, the range of available economic and labour market indicators is more restricted at a sub-national level, preventing us from delving deeper into different possible links between economic downturn and fertility. In addition, some survey-based indicators turned out to be too uncertain and unstable at the level of NUTS-2 regions. Third, our study does not consider the role of education as a potent moderator of the association between adverse economic circumstances and fertility trends. Clearly, the idea that all social groups are equally vulnerable to economic uncertainty is both logically thin and empirically tenuous (Kreyenfeld 2015). Finally, the role of changing labour market conditions during the *Great Recession* cannot be solely ascribed to (long-term) unemployment. In many countries, an increasing number of people—the emerging class of “precariat”—are facing uncertain lives, moving in and out of low-paid “stopgap” jobs that may give little meaning to their lives (Standing 2011). Like in the USA (Seltzer 2019), this group might have contributed strongly to the unexpected and puzzling continuation of fertility decline during the period of post-recession recovery in 2014–2018 in many European countries including the Nordic countries (Comolli 2019) and most of Western Europe (except Austria, Germany and Switzerland) (VID 2018), which took place during the time of declining unemployment and solid economic growth.

Economic uncertainty has become an intrinsic feature of contemporary globalizing world, and the relationship between economic conditions and family dynamics is to remain a major topic of public interest in the years to come. The challenge for future research will be to integrate better macro- and micro-level evidence on the impact of economic uncertainty on family behaviour and to address the economic uncertainty/fertility nexus from a life-course perspective, considering a broader set of social, economic, policy and cultural factors.

### Electronic supplementary material

Below is the link to the electronic supplementary material.

Supplementary material 1 (DOCX 31 kb)

Supplementary material 2 (XLSX 46 kb)

## Figures and Tables

**Fig. 6:**
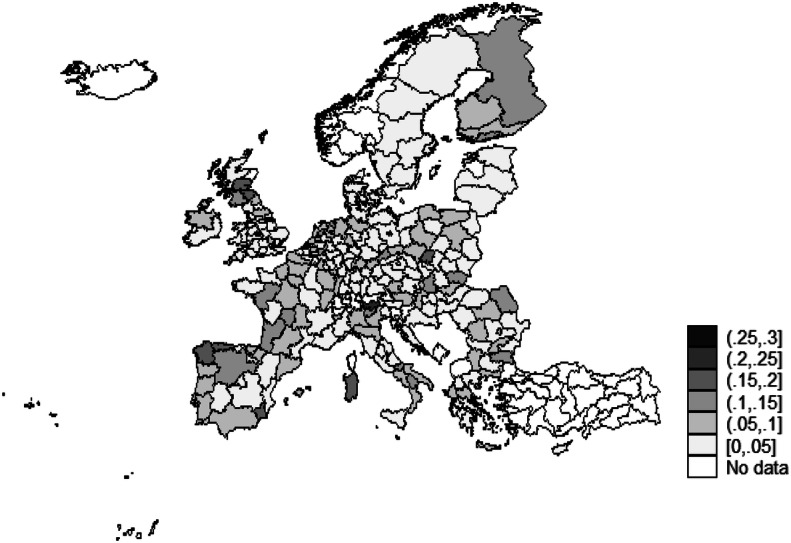
Absolute differences between the actual and predicted TFR (under S5), NUTS-2 averages over 2002–2014

## Data Availability

The datasets analysed during the current study are available from the corresponding author on request.

## Appendix

### Appendix 1: NUTS-2 regions excluded from the analysis

Out of the 276 NUTS-2 regions in the European Union as of January 2017, 24 regions were excluded from our analysis and two regions of Slovenia were replaced by one region covering the whole country, leaving our analysed sample with 251 regions.

The list of excluded regions is as follows:

1. Overseas territories with distinct socioeconomic set up, not directly comparable to European regions (10 regions).
  - France (5 regions): NUTS-2 regions covering French overseas departments: Guadeloupe, Martinique, Guyane, La Réunion and Mayotte.
  - Portugal (2 regions): Azores (*Região Autónoma dos Açores*) and Madeira (*Região Autónoma da Madeira*).
  - Spain (3 regions): Ceuta (*Ciudad Autónoma de Ceuta*), Melilla (*Ciudad Autónoma de Melilla*) and Canary Islands (*Canarias*).
2. Regions with important boundary shifts during the analysed period (4 regions).
  - Slovenia (2 regions): due to boundary shifts in 2006 between the two NUTS-2 regions in the country (eastern Slovenia and western Slovenia), we did not analyse these regions separately and considered the whole country as one region.
  - UK (2 regions): two regions with boundary changes in 2010: Cheshire and Merseyside.
3. Regions with missing time-series data on labour market indicators or GDP for the analysed period (12 regions).
  - Austria (4 regions): Burgenland, Carinthia (*Kärnten*), Salzburg and Vorarlberg.
  - Finland (1 region): Åland islands.
  - France (1 region): Corsica (*Corse*).
  - UK (6 regions): Inner London West, Inner London East, Outer London East and North East, Outer London South, Outer London West and North West, North Eastern Scotland, Highlands and Islands.

### Appendix 2

See Table 1.

**Table 1:** Sample description: sample size, reliability and completeness, mean and standard deviation of the economic and fertility indicators for 251 NUTS-2 regions, 2001–2014

Indicator (1)	Years (2)	N (3)	% with low reliability^a^ (4)	% missing before imputation^b^ (5)	% missing after imputation (6)	Mean (7)	St dev (8)
TFR	2002–2014	3263	NA	1.3	1.3	1.54	0.270
ASFR 15–19	2002–2014	3263	NA	3.3	3.3	0.069	0.049
ASFR 20–24	2002–2014	3263	NA	3.3	3.3	0.256	0.087
ASFR 25–29	2002–2014	3263	NA	3.3	3.3	0.479	0.118
ASFR 30–34	2002–2014	3263	NA	2.9	2.9	0.471	0.113
ASFR 35+	2002–2014	3263	NA	3.3	3.3	0.264	0.090
Unemployment rate 20–64	2001–2013	3263	4.8%	3.3	2.7	8.3	4.9
Unemployment rate 15–24	2001–2013	3263	23.6%	10.3	6.8	20.2	11.2
Unemployment rate 25+	2001–2013	3263	7.3%	3.6	3.0	7.4	4.4
% long-term unemployment	2001–2013	3263	22.3%	11.1	8.8	40.3	13.6
% NEET	2001–2013	3263	11.7%	4.8	4.1	15.6	7.0
GDP growth	2001–2013	3263	NA	1.0	1.0	1.2	4.2

^a^The number of observations the value is based on exceeds the first but not the second reliability threshold of Eurostat (the indicator is published but characterized by low reliability). The reliability analysis is based on data downloaded in March 2016. ^b^Missing values may be due to low reliability (the number of observations the value is based on does not exceed the first reliability threshold of Eurostat)

### Appendix 3

See Table 2.

**Table 2:** Main model estimates as specified by Eq. (3), EU-28 2001–2014

	TFR	ASFR 15–19	ASFR 20–24	ASFR 25–29	ASFR 30–34	ASFR 35+
*Calendar time (piecewise linear spline)*						
2002–2007	0.0236***	− 0.000610**	− 0.00405***	0.00338**	0.0149***	0.0111***
	(0.00367)	(0.000292)	(0.00123)	(0.00156)	(0.00174)	(0.000954)
2008–2010	− 0.00825**	− 0.00272***	− 0.00864***	− 0.00809***	0.00122	0.00799***
	(0.00402)	(0.000451)	(0.00101)	(0.00196)	(0.00156)	(0.000828)
2011–2014	0.00196	− 0.00125**	− 0.00420***	− 0.00103	0.00347	0.00731***
	(0.00653)	(0.000576)	(0.00141)	(0.00219)	(0.00216)	(0.00117)
*Unemployment rate*						
Within-region effect	− 0.00432***	− 0.000137**	− 0.000331***	− 0.00125***	− 0.000481	− 0.000547*
	(0.00148)	(0.0000577)	(0.000118)	(0.000441)	(0.000643)	(0.000325)
Between-region within-country effect	− 0.00293	− 0.00140***	− 0.00428***	0.0000126	− 0.00172	− 0.00148
	(0.00399)	(0.000481)	(0.000998)	(0.00202)	(0.00139)	(0.00164)
Between-country effect	− 0.0200	− 0.00345***	− 0.00304	0.00369	0.00405	− 0.000182
	(0.0154)	(0.00101)	(0.00191)	(0.00357)	(0.00341)	(0.00276)
*Long-term unemployment*						
Within-region effect	− 0.000959***	− 0.0000416	− 0.000143**	− 0.000254***	− 0.000298***	− 0.000150***
	(0.000241)	(0.0000392)	(0.0000692)	(0.0000894)	(0.0000887)	(0.0000577)
Between-region within-country effect	− 0.00381***	0.000187	0.000230	− 0.00150**	− 0.000957**	− 0.000260
	(0.00131)	(0.000253)	(0.000526)	(0.000668)	(0.000452)	(0.000545)
Between-country effect	− 0.00816**	0.0000776	0.000783	− 0.000970	− 0.00586***	− 0.00393***
	(0.00386)	(0.000525)	(0.000996)	(0.00156)	(0.00166)	(0.00117)
*GDP decline*						
Within-region effect	− 0.000181	− 0.0000960**	− 0.0000699	0.000155	− 0.0000127	− 0.000111
	(0.000377)	(0.0000459)	(0.0000916)	(0.000128)	(0.000181)	(0.000113)
Between-region within-country effect	0.00173	0.00466**	0.0115**	0.00517	− 0.0135***	− 0.0155***
	(0.0120)	(0.00219)	(0.00456)	(0.00617)	(0.00417)	(0.00504)
Between-country effect	0.0164	− 0.0118***	− 0.0287***	0.00959	0.0375***	− 0.0187***
	(0.0207)	(0.00295)	(0.00555)	(0.00857)	(0.00912)	(0.00647)
*% NEET*						
Within-region effect	− 0.000439	− 0.000122	− 0.000166	0.000253*	− 0.000292*	− 0.000239*
	(0.000451)	(0.000158)	(0.000208)	(0.000141)	(0.000169)	(0.000130)
Between-region within-country effect	0.00916***	0.00458***	0.0102***	0.00261**	− 0.00192**	− 0.00100
	(0.00265)	(0.000580)	(0.00120)	(0.00130)	(0.000882)	(0.00106)
Between-country effect	0.00310	0.00760***	0.00405	− 0.00656*	0.0000708	0.00292
	(0.0101)	(0.00163)	(0.00308)	(0.00391)	(0.00415)	(0.00294)
*Constant*	1.428***	0.0723***	0.279***	0.480***	0.402***	0.195***
	(0.0372)	(0.00527)	(0.00992)	(0.0153)	(0.0166)	(0.0115)
*Random terms*						
Country: sd(2002–2007)	0.0177***	0.00116***	0.00589***	0.00761***	0.00862***	0.00455***
	(0.00280)	(0.000232)	(0.000909)	(0.00127)	(0.00126)	(0.000722)
Country: sd(2008–2010)	0.0185***	0.00190***	0.00424***	0.00934***	0.00714***	0.00356***
	(0.00339)	(0.000371)	(0.000897)	(0.00169)	(0.00133)	(0.000697)
Country: sd(2011–2014)	0.0330***	0.00267***	0.00666***	0.0108***	0.0107***	0.00562***
	(0.00477)	(0.000442)	(0.00105)	(0.00164)	(0.00164)	(0.000922)
Country: sd (unempl rate)	0.00620***	0.000171***	0.000356***	0.00143***	0.00269***	0.00121***
	(0.00124)	(0.0000526)	(0.000107)	(0.000453)	(0.000560)	(0.000270)
Country: sd (% Long-term unempl)	0.000872***	0.000150***	0.000242***	0.000292***	0.000291***	0.000191***
	(0.000222)	(0.0000363)	(0.0000700)	(0.0000915)	(0.0000896)	(0.0000542)
Country: sd(GDP growth)	0.00153***	0.000163***	0.000332***	0.000460***	0.000779***	0.000484***
	(0.000441)	(0.0000426)	(0.0000827)	(0.000151)	(0.000200)	(0.000113)
Country: sd(%NEET)	0.00105***	0.000667***	0.000681***	1.08e−10***	0.000345***	0.000349***
	(0.000919)	(0.000133)	(0.000196)	(5.55e–10)	(0.000278)	(0.000159)
Country: sd(_cons)	0.171***	0.0236***	0.0430***	0.0688***	0.0773***	0.0512***
	(0.0254)	(0.00367)	(0.00671)	(0.0103)	(0.0113)	(0.00888)
Region: sd(_cons)	0.0642***	0.0148***	0.0319***	0.0490***	0.0316***	0.000000143
	(0.0339)	(0.00155)	(0.00220)	(0.00242)	(0.00177)	(0.0000320)
Residual: rho1	0.575***	0.660***	0.866***	0.646***	0.454***	0.601***
	(0.0247)	(0.0265)	(0.0232)	(0.0266)	(0.0254)	(0.0188)
Residual: rho2	0.354***	0.239***			0.290***	0.382***
	(0.0263)	(0.0276)			(0.0276)	(0.0189)
Residual: rho3	0.00661					
	(0.0342)					
Residual: sd	0.0833***	0.0106***	0.0211***	0.0180***	0.0182***	0.0426***
	(0.0264)	(0.00178)	(0.00183)	(0.000659)	(0.00104)	(0.00193)
N	2928	2827	2827	2865	2865	2865
AIC	− 10388.8	− 20694.0	− 16605.1	− 15113.3	− 15406.8	− 17499.7
BIC	− 10215.3	− 20527.5	− 16444.5	− 14952.4	− 15239.9	− 17332.8
ll	5223.4	10375.0	8329.6	7583.7	7731.4	8777.8
k	29	28	27	27	28	28

### Appendix 4

See Table 3.

**Table 3:** Heterogeneity of the within-region effects, EU-28 2001–2014

	Main model + interaction with recession dummy	Main model + interaction with a trend dummy	Main model + interaction with a level dummy
*Calendar time (piecewise linear spline)*			
2002–2007	0.024927***	0.0237929***	0.024175***
	(0.0038738)	(0.0036764)	(0.0036664)
2008–2010	− 0.01061**	− 0.0090365**	− 0.00849**
	(0.0042918)	(0.0040769)	(0.0039987)
2011–2014	0.002334	0.0021706	0.001062
	(0.0064797)	(0.0065965)	(0.0066161)
*Recession (ref: year: < 2009)*			
year ≥ 2009	0.002377		
	(0.0041981)		
*Unemployment rate*			
Within-region effect	− 0.00295*	− 0.0034862**	− 0.00587***
	(0.0015673)	(0.0015777)	(0.0016904)
Within-region effect * recession (year >=2009)	− 0.00102		
	(0.0010005)		
Dummy: upward trend		0.0057797***	
		(0.0020736)	
Dummy: above the regional average			0.003494*
			(0.002101)
Within-region effect * dummy: upward trend		− 0.0006678	
		(0.0005567)	
Within-region effect * dummy: above regional average			0.001975
			(0.0012541)
Between-region within-country effect	− 0.00306	− 0.0027896	− 0.00324
	(0.0040038)	(0.0040058)	(0.0039933)
Between-country effect	− 0.0209	− 0.0197472	− 0.0199
	(0.0153925)	(0.0152972)	(0.0152634)
*Long-term unemployment*			
Within-region effect	− 0.00072***	− 0.0012355***	− 0.0014***
	(0.0002653)	(0.000264)	(0.0003148)
Within-region effect * recession (year >=2009)	− 0.00049*		
	(0.0002562)		
Dummy: upward trend		− 0.007072***	
		(0.0018821)	
dummy: above the regional average			− 0.00213
			(0.0019525)
Within-region effect * dummy: upward trend		0.0007957***	
		(0.0001828)	
Within-region effect * dummy: above regional average			0.001164***
			(0.0003477)
Between-region within-country effect	− 0.00382***	− 0.0037551***	− 0.00376***
	(0.0013173)	(0.0013181)	(0.0013126)
Between-country effect	− 0.00831**	− 0.0083057**	− 0.00827**
	(0.0038631)	(0.0038378)	(0.0038289)
*GDP decline*			
Within-region effect	0.00023	0.0001353	0.00067
	(0.0005266)	(0.0004285)	(0.0004938)
Within-region effect * recession (year >=2009)	− 0.001195***		
	(0.0004234)		
dummy: downward trend		0.000632	
		(0.0019626)	
dummy: above the regional average			− 0.00117
			(0.001866)
Within-region effect * dummy: downward trend		− 0.0006909	
		(0.0004842)	
Within-region effect * dummy: above regional average			− 0.00138**
			(0.0005463)
Between-region within-country effect	− 0.00229	− 0.0017299	− 0.00128
	(0.01205)	(0.0120609)	(0.0120058)
Between-country effect	− 0.0196	− 0.0172393	− 0.01696
	(0.0206812)	(0.0205532)	(0.020507)
*% NEET*			
Within-region effect	0.000603	− 0.0001493	− 0.00183***
	(0.0005197)	(0.0005306)	(0.0006268)
Within-region effect * recession (year >= 2009)	− 0.00209***		
	(0.0006012)		
Dummy: upward trend		0.0016945	
		(0.0018891)	
dummy: above the regional average			0.003454*
			(0.00191)
Within-region effect * dummy: upward trend		− 0.0003026	
		(0.0003871)	
Within-region effect * dummy: above regional average			0.001916**
			(0.0007642)
Between-region within-country effect	0.009168***	0.0090588***	0.009205***
	(0.0026573)	(0.002659)	(0.0026477)
Between-country effect	0.003149	0.0033	0.00257
	(0.0101323)	(0.0100715)	(0.0100456)
*Constant*	1.426934***	1.42915***	1.419569***
	(0.037307)	(0.0371026)	(0.0370543)
*Random terms*			
Country: sd(2002–2007)	0.018803***	0.0177335***	0.017689***
	(0.0029532)	(0.0028215)	(0.0027983)
Country: sd(2008–2010)	0.018953***	0.0186918***	0.01835***
	(0.003397)	(0.0033891)	(0.0033683)
Country: sd(2011–2014)	0.032705***	0.0332938***	0.033404***
	(0.0047231)	(0.0048053)	(0.0048316)
Country: sd(unempl rate)	0.006208***	0.0065097***	0.006303***
	(0.0012164)	(0.0013046)	(0.0012164)
Country: sd(% Long-term unempl)	0.00085***	0.0009126***	0.000917***
	(0.000223)	(0.000234)	(0.0002278)
Country: sd(GDP growth)	0.001848***	0.001252***	0.00128***
	(0.0004552)	(0.0004085)	(0.0004311)
Country: sd(%NEET)	0.000989	0.0012329	0.000624
	(0.0006962)	(0.0009826)	(0.000684)
Country: sd(_cons)	0.171172***	0.1700037***	0.1697***
	(0.0254925)	(0.0253053)	(0.0252653)
Region: sd(_cons)	0.054976	0.0000165	0.068735***
	(0.0609256)	(0.0079738)	(0.0245518)
Residual: rho1	0.555403***	0.5644423***	0.569057***
	(0.0241198)	(0.0222139)	(0.0243553)
Residual: rho2	0.393828***	0.3786001***	0.361989***
	(0.0258125)	(0.0241194)	(0.0262262)
Residual: rho3	− 0.00208	0.0195609	− 0.00195
	(0.0380524)	(0.0213764)	(0.0334591)
Residual: sd	0.089827**	0.1055487***	0.079331***
	(0.0378324)	(0.0045159)	(0.0213188)
N	2928	2928	2928
AIC	− 10428.9	− 10423.05	− 10408.4
BIC	− 10225.5	− 10201.72	− 10187.1
ll	5248.454	5248.527	5241.218
k	34	37	37

### Appendix 5

See Fig. 6.

## Footnotes

1 We also considered using indicators of economic deprivation and social exclusion (i.e. severe material deprivation, the proportion of people at risk of poverty and social exclusion and at-risk-of-poverty rate). However, we did not include them as they are not available at the NUTS-2 level for the whole analysed period and they are based on small survey samples, suffering considerable year-to-year fluctuations.

2 This is probably the most common lag, accounting for the duration of pregnancy (around 9 months) and a few months’time for women and men to react to changing economic conditions. Individual studies apply a time lag typically ranging up to 2 years. Aggregate correlations between GDP growth and fertility change across highly developed countries betwen 1956 and 2008 were much stronger when one-year lag rather than two-year lag was applied (Sobotka et al 2011: 294, Table A1). However, for individual countries, two-year lags often gave stronger correlation than one-year lag (ibid., Table A2).

3 It was not possible to estimate a model with all random effects and their correlations. We have, however, estimated models with several combinations of random effects and the correlations were mostly insignificant. We also examined the AIC and BIC of the models with and without the correlations and the two statistics clearly suggested to use the more parsimonious models.

4 To construct this variable, we smoothed the economic indicators to extract the trend and get rid of year-to-year fluctuations in economic indicators.

5 The exceptions were the models for ASFR 20–24 and 30–34, in which the GDP decline at *t*-2 turned out to predict fertility better than the GDP decline at *t*-1, but at the same time exactly the reverse was observed in the model for ASFR 25–29.
