# Supplementary material for: The Great Recession and Fertility in Europe: A Sub-national Analysis
Source: Eur J Popul. 2020 Apr 3;37(1):29–64. doi: 10.1007/s10680-020-09556-y (PMC7864853; doi:10.1007/s10680-020-09556-y)
Supplement: Supplementary file 1 — Supplementary material 1 (DOCX 31 kb) [file 10680_2020_9556_MOESM1_ESM.docx]

**ONLINE SUPPLEMENTARY MATERIAL**

**Appendix 1.**

Economic indicators used in our model can be strongly correlated, which may lead to multi-collinearity problems. Multi-collinearity means that the estimated coefficients are unreliable and unstable. One method of testing for multi-collinearity is to compute Variance Inflation Factors (VIF). (VIF-1)*100% tell us to what extent is the variance of the coefficient higher than it would be if the coefficient was completely unrelated to the remaining covariates in the model. The problem with this method is that there is no consensus about the upper threshold of VIF which would suggest multi-collinearity problems. Some researchers use the upper limit of 10, other use 5 and yet other ones even 2.5. They agree, however, that high VIF is not problematic when it concerns control variables (see, for instance, the blog of Paul Allison on Statistical Horizons <https://statisticalhorizons.com/multicollinearity>). In our models, the VIF is mostly lower than 2.5 for all major explanatory covariates, i.e., those which refer to within region effects, and certainly does not exceed 3, which we take as a good sign. The VIFs from our models are displayed in Table 1.

**Table 1. Variance Inflation Factors for covariates in our models**

| **Dependent variable**  **TFR** | | | **Dependent variables**  **ASFR 15-19 and 20-24** | | | **Dependent variables**  **ASFR 25-29, 30-34, 35+** | | |
| --- | --- | --- | --- | --- | --- | --- | --- | --- |
| Variable | VIF | 1/VIF | Variable | VIF | 1/VIF | Variable | VIF | 1/VIF |
| year2009-10 | 3.2 | 0.31 | unempl_15-24_BRWC | 4.23 | 0.24 | unempl_25-64_BRWC | 9.28 | 0.11 |
| unempl_20-64_BRWC | 2.64 | 0.38 | neets_BC | 3.8 | 0.26 | unempl_25-64_BC | 7.96 | 0.13 |
| neets_BRWC | 2.62 | 0.38 | neets_BRWC | 3.64 | 0.27 | year2009-10 | 3.1 | 0.32 |
| unempl_20-64_WR | 2.34 | 0.43 | year2009-10 | 3.11 | 0.32 | unempl_25-64_WR | 2.2 | 0.46 |
| year2011-14 | 2.01 | 0.50 | unempl_15-24_WR | 2.95 | 0.34 | neets_BRWC | 2.05 | 0.49 |
| neets_BC | 1.96 | 0.51 | unempl_15-24_BC | 2.69 | 0.37 | neets_BC | 2.02 | 0.50 |
| neets_WR | 1.96 | 0.51 | neets_WR | 2.35 | 0.43 | year2011-14 | 1.93 | 0.52 |
| unempl_lt_BRWC | 1.86 | 0.54 | unempl_lt_BRWC | 2.11 | 0.47 | neets_WR | 1.84 | 0.54 |
| year2001-08 | 1.85 | 0.54 | year2011-14 | 1.97 | 0.51 | year2001-08 | 1.83 | 0.55 |
| unempl_lt_WR | 1.65 | 0.61 | year2001-08 | 1.84 | 0.54 | unempl_lt_BRWC | 1.67 | 0.60 |
| unempl_20-64_BC | 1.61 | 0.62 | unempl_lt_BC | 1.66 | 0.60 | unempl_lt_BC | 1.63 | 0.61 |
| unempl_lt_BC | 1.58 | 0.63 | unempl_lt_WR | 1.55 | 0.65 | unempl_lt_WR | 1.63 | 0.61 |
| gdp_WR | 1.45 | 0.69 | gdp_WR | 1.42 | 0.70 | gdp_WR | 1.46 | 0.69 |
| gdp_BRWC | 1.34 | 0.75 | gdp_BRWC | 1.4 | 0.71 | gdp_BRWC | 1.32 | 0.76 |
| gdp_BC | 1.09 | 0.92 | gdp_BC | 1.11 | 0.90 | gdp_BC | 1.1 | 0.91 |

Note: WR means within region, BRWC means between region within country and BC stands for between country.

**Appendix 2.**

Our model is quite complex. We have therefore tested whether a more parsimonious model fits the data at least equally well. The models we tested are presented below and the AIC and BIC values is displayed in Table 1 in Appendix 1. The comparison of AIC and BIC of these models clearly shows that the model M0, used in the paper, fits the data best.

M4: random-intercept model

${FR}_{crt}={(\beta}_{0}+\mu_{0c}+\mu_{0r})+\sum_{j=1}^{m} \beta_{j}\cdot t+{\sum_{i=1}^{n} \gamma_{i}\cdot X_{icrt-1}}+\varepsilon_{crt}$

M3: fixed-effects model

${FR}_{crt}=\sum_{j=1}^{m} \beta_{j}\cdot(t-\bar{t})+{\sum_{i=1}^{n} \gamma_{i}\cdot\left( X_{icrt-1}-\bar{X_{icr\cdot}} \right)}]+(\varepsilon_{crt}-\bar{\varepsilon_{cr\cdot}})$

M2: random-intercept model with a decomposition into within-region, between-region within-country and between-country effects

${FR}_{crt}={(\beta}_{0}+\mu_{0c}+\mu_{0r})+\sum_{j=1}^{m} \beta_{j}\cdot t+{\sum_{i=1}^{n} {{[\gamma}^{WR}}_{i}\cdot\left( X_{icrt-1}-\bar{X_{icr\cdot}} \right)+{\gamma^{BRWC}}_{i}\left( \bar{X_{icr\cdot}}-\bar{X_{ic\cdot\cdot}} \right)+{\gamma^{BC}}_{i}\bar{X_{ic\cdot\cdot}}}]+\varepsilon_{crt}$

M1: model with random slopes on the time trend and a decomposition into within-region, between-region within-country and between-country effects

$${FR}_{crt}={(\beta}_{0}+\mu_{0c}+\mu_{0r})+\sum_{j=1}^{m} \left( \beta_{j}+\mu_{jc} \right)\cdot t+{\sum_{i=1}^{n} {{[\gamma}^{WR}}_{i}\cdot\left( X_{icrt-1}-\bar{X_{icr\cdot}} \right)+{\gamma^{BRWC}}_{i}\left( \bar{X_{icr\cdot}}-\bar{X_{ic\cdot\cdot}} \right)+{\gamma^{BC}}_{i}\bar{X_{ic\cdot\cdot}}}]+\varepsilon_{crt}$$

M0: model with random slopes on the time trend and within-region effects and a decomposition into within-region, between-region within-country and between-country effects (model presented in the paper)

$${FR}_{crt}={(\beta}_{0}+\mu_{0c}+\mu_{0r})+\sum_{j=1}^{m} \left( \beta_{j}+\mu_{jc} \right)\cdot t+{\sum_{i=1}^{n} \left[ ({\gamma^{WR}}_{i}+\mu_{ic} \right)\cdot\left( X_{icrt-1}-\bar{X_{icr\cdot}} \right)+{\gamma^{BRWC}}_{i}\left( \bar{X_{icr\cdot}}-\bar{X_{ic\cdot\cdot}} \right)+{\gamma^{BC}}_{i}\bar{X_{ic\cdot\cdot}}}]+\varepsilon_{crt}$$

Table 2. Comparison of the data fit of the model M0, used in the paper, to the data fit of more parsimonious models

|  |  |  |  |  |  |
| --- | --- | --- | --- | --- | --- |
|  | **M0** | **M1** | **M2** | **M3** | **M4** |
| **TFR** |  |  |  |  |  |
| N | 2929 | 2929 | 2929 | 2929 | 2929 |
| AIC | -10392.1 | -10258.0 | -9565.2 | -8684.3 | -9545.5 |
| BIC | -10218.6 | -10108.5 | -9433.6 | -8636.4 | -9461.8 |
| **ASFR 15-19** |  |  |  |  |  |
| N | 2827 | 2827 | 2827 | 2827 | 2827 |
| AIC | -20693.9 | -20615.3 | -20346.5 | -19958.7 | -20175.8 |
| BIC | -20527.4 | -20472.5 | -20227.5 | -19911.1 | -20104.4 |
| **ASFR 20-24** |  |  |  |  |  |
| N | 2827 | 2827 | 2827 | 2827 | 2827 |
| AIC | -16605.0 | -16541.2 | -16037.2 | -14421.0 | -15932.2 |
| BIC | -16444.5 | -16404.4 | -15924.2 | -14373.4 | -15860.8 |
| **ASFR25-29** |  |  |  |  |  |
| N | 2865 | 2865 | 2865 | 2865 | 2865 |
| AIC | -15115.3 | -15085.6 | -14605.6 | -13938.8 | -14598.2 |
| BIC | -14960.3 | -14948.5 | -14492.4 | -13891.1 | -14526.6 |
| **ASFR 30-34** |  |  |  |  |  |
| N | 2865 | 2865 | 2865 | 2865 | 2865 |
| AIC | -15406.0 | -15321.5 | -14617.9 | -13694.4 | -14584.0 |
| BIC | -15239.1 | -15178.4 | -14492.7 | -13646.7 | -14512.5 |
| **ASFR 35+** |  |  |  |  |  |
| N | 2865 | 2865 | 2865 | 2865 | 2865 |
| AIC | -17501.4 | -17404.7 | -16948.8 | -16066.9 | -16934.5 |
| BIC | -17340.5 | -17267.7 | -16823.6 | -16019.2 | -16863.0 |

**Appendix 3.**

In our model used in the paper all economic indicators are lagged by one time unit (one year) in order to account for the fact that conception takes place 9 months before the birth. We have, however, verified whether lagging our economic indicators by one year is sufficient to capture fertility reactions to changing economic conditions. To this end, we assessed two-year lagged values of economic indicators in addition to one year lags. This robustness check was performed on a slightly simplified version of our model (i.e., model M2 as specified in Appendix 3 in the Online Supplementary Material), because adding random slopes on our economic indicators lagged by one and two years would result in an extremely complex model and convergence problems. The findings of this robustness check are presented in tables in supplementary Excel file (sheet Appendix 2). Even though changes in some of the economic indicators lagged by two years turned out to be significantly related to fertility (this was mainly the case of unemployment rate), introducing them into the model did not change the significance or the direction of the estimated coefficients at one year lag in the vast majority of cases^[[1]](#footnote-1)^. Furthermore, the BIC statistic clearly showed that the more parsimonious models, with one year lags, had a better fit.

**Appendix 4.**

The data used in our study contained some missing values, which we imputed with a cubic spline interpolation. We performed a robustness check in order to verify whether the imputation did not affect our findings. To this end, we re-estimated our models using the pre-imputation dataset. These findings are presented in tables in supplementary Excel file (sheet Appendix 4). Hardly any changes have been observed in our findings.

1. The exceptions were the models for ASFR 20-24 and 30-34, in which the GDP decline at t-2 turned out to predict fertility better than the GDP decline at t-1, but at the same time exactly the reverse was observed in the model for ASFR 25-29. [↑](#footnote-ref-1)
